# Evaluating the Effect of Simulation‐Based Handover Training and Its Predictors: A Quasiexperimental Study

**DOI:** 10.1155/jonm/6626003

**Published:** 2026-07-29

**Authors:** Kexin Tan, Jack Pun

**Affiliations:** ^1^ Department of English and Communication, The Hong Kong Polytechnic University, 11 Yuk Choi Rd, Hung Hom, Kowloon, Hong Kong SAR, China, polyu.edu.hk; ^2^ Department of English, The Chinese University of Hong Kong, Shatin, New Territories, Hong Kong SAR, China, cuhk.edu.hk

**Keywords:** clinical competence, nursing education, patient handoff, resilience, simulation training

## Abstract

**Aim(s):**

This study aimed primarily to evaluate the effect of simulation‐based handover training on nurses’ perceived handover competency (PC). A secondary aim was to identify demographic and psychological factors that predict nurses’ perceived handover competency.

**Design:**

A single‐group, quasi‐experimental, pretest–posttest design was adopted.

**Methods:**

Using cluster sampling and purposive sampling, 558 qualified, English‐proficient nurses were recruited from 29 hospitals in Hong Kong. A 4‐h simulation‐based training on the CARE protocol was delivered. Validated instruments with high reliability (overall Cronbach’s *α* = 0.954) measured perceived competency and four psychological factors immediately pre‐ and post‐intervention. The primary objective, assessing changes in perceived handover competency (PC), was analyzed using paired‐samples *t*‐tests. The second objective, exploring the predictors of PC, was evaluated using Pearson’s correlation and ordinary least squares (OLS) regression. Ethical approval and written consent were obtained. Reporting followed the TREND statement.

**Results:**

The simulation‐based training demonstrated a large positive effect on nurses’ perceived handover competency (Cohen’s *d* = 1.198, *p* < 0.001). Staff position (*F* = 3.813) and age group (*F* = 3.204) were the only demographic factors associated with significant increases in PC, with registered nurses and nurses aged 30–34 years showing the greatest gains. Perceived competency correlated strongly with resilience (*r* = 0.920, *p* < 0.001). Baseline demographic and professional factors explained only 1.8% of the variance in perceived competency (*R* ^2^ = 0.018, *p* > 0.05), whereas internal psychological factors increased the explained variance to 76.5% (*R* ^2^ = 0.765, *p* < 0.001). Resilience (*β* = 0.376, *p* < 0.001) and intention to complete handover (*β* = 0.320, *p* < 0.001) were the strongest predictors.

**Conclusion:**

Simulation‐based training is a highly effective intervention for enhancing nursing handover competency. Perceived handover competency was mainly predicted by internal psychological attributes rather than professional factors. Educational programs must prioritize soft skills, specifically resilience, to optimize clinical communication.

**Implication for Nursing Management:**

For nurse education, the findings indicate that communication training should shift from purely technical training to integrating resilience‐building strategies and psychological skills. In clinical practice, nurse leaders should implement ongoing mentorship and feedback to help nurses fully internalize handover protocols. Regarding healthcare policy and management, the findings indicate that hospital administrators should design separate training tracks tailored to the different experience levels of junior and senior nurses, maximizing the growth potential of each group.

## 1. Introduction

Nursing handover, or the process of transferring patient information, responsibility, and accountability, is critical for ensuring patient safety and continuity of care [1]. As a routine yet high‐stakes practice, it directly influences clinical decision‐making, care prioritization, and interdisciplinary coordination [2]. Poor handover practices have been consistently linked to adverse patient outcomes, including medication errors, delayed treatments, and preventable complications [3, 4]. Effective nursing handover is characterized by clarity, completeness, and mutual understanding [5], yet achieving consistent practice remains a global challenge [4]. Structured handover protocols, such as Connect, Ask, Respond, and Empathize (CARE), have been adopted internationally to standardize handover content and interactions to improve information quality and communication [6]. Within this framework, Connect requires nurses to establish a safe, trusting, and respectful environment. Ask entails gathering necessary medical details for nursing handover. Respond means providing clear, relevant explanations and acting on the information gathered during nursing handover. Empathize refers to acknowledging patients’ feelings, comforting them, and respective their needs for privacy and involvement [7]. Training in structured protocols has been shown to improve perceived handover competence [7–9].

A growing body of literature has indicated that the efficacy of handover training depends not only on curricular design but also on who participates and how they engage with the content [10]. Current evidence shows that nursing handover competency is influenced by a range of factors, including handover education and clinical experience [11, 12]. Professional factors such as the route of initial training may also influence how nurses engage with new handover curricula and their competence in applying handover protocols [11, 13]. In contrast, demographic characteristics such as age, gender, and education level often exhibit weaker or more context‐dependent associations, frequently acting as moderators rather than primary predictors of performance [11, 12].

Psychological internal factors are increasingly recognized as the proximal predictors of behavioral change in nursing education. Central to these internal factors is the concept of self‐efficacy, or the belief in one’s ability to successfully execute specific tasks [14]. Recent scholarship has consistently highlighted self‐efficacy as a core outcome of handover education. For instance, Lee and Lim [15] found that a simulation‐based handover program significantly increased nursing students’ self‐efficacy, suggesting that immersive practice bridges the gap between theoretical knowledge and the confidence to perform. Similarly, Chung et al. utilized a randomized controlled trial (RCT) to demonstrate that blended learning programs not only improve self‐efficacy but also directly enhance communication skill competence [16]. Gheisari et al. further expanded on this by showing that clinical supervision models can bolster nurses’ self‐efficacy specifically during the handover process in medical and surgical wards [17]. Moreover, Oh identified that posttraining self‐efficacy is affected by a nurse’s communication competence and their prior experience with handover [13].

However, despite these foundational works, important gaps remain in the literature. Traditionally, handover research has focused on measuring perceived handover competency (PC) or general self‐efficacy as primary indicators of training effects [10, 16]. While evaluating these broad outcomes is valuable for establishing baseline efficacy, treating perceived competency as a monolithic endpoint obscures the underlying mechanisms that drive it. First, there is a lack of evidence regarding how demographic and professional factors shape nurses’ perceived competency after handover training. Second, and more importantly, no study to date has offered a nuanced examination of the specific behavioral and psychological dimensions that influence this perceived competency. Little research has explored how motivational factors (operationalized here as the intention to initiate and complete a handover) and volitional coping mechanisms (operationalized here as resilience in overcoming distractions and difficulties during a handover) interact to shape a nurse’s perceived handover competency.

This study proposes that predicting perceived competency requires deconstructing self‐efficacy through the lens of Kuhl’s action control theory [18]. According to this theory, successful behavioral change requires not only selective motivation but also volitional control. The first stage, selective motivation, is represented by intention to initiate handover (IIH) and intention to complete handover (ICH) in this study [18]. The second stage of the theory, volitional control, is where resilience in handover (RH) becomes essential [18, 19]. Resilience is understood in the nursing discipline as the ability to adapt and function optimally despite adverse conditions [20]. In the context of handover, it can refer to the ability to identify and intercept potential errors during the transfer of care. Resilience serves as a protective mechanism, shielding a learner’s original intention from potential distractions, such as hierarchical pressure, interruptions, and high cognitive demands [18, 19]. Under this unified framework, intentions provide the energy to set nursing goals, while resilience provides the persistence to strive towards them. When combined with the nurse’s perceptions of protocols (PP) [8], these factors create a comprehensive psychological infrastructure that dictates whether training translates into real‐world perceived competency.

This study fills a significant gap in the literature by providing empirical evidence of how internal psychological factors shape perceived handover competency. The need to study perceived competency through the lens of action control is particularly relevant in high‐pressure clinical settings such as Hong Kong. Nursing practice in Hong Kong is often characterized by a strict hierarchical medical culture and high power distance, which can affect junior nurses’ intention to initiate communication [21]. Identifying the internal factors that affect nurses’ perceived competency in this context allows for the development of more targeted, culturally sensitive, and resilient safety cultures.

The first objective of this study was to evaluate the effect of a simulation‐based training intervention on nurses’ handover performance by measuring changes in perceived handover competency (PC). A second objective was to explore how demographic, professional, and intrinsic psychological factors moderate the training’s effect and predict improvements in perceived competency. This research aimed to answer the following questions:

 RQ1: To what extent does the CARE protocol‐related simulation‐based training affect the perceived handover competency (PC) of nurses from pretest to posttest?
 RQ2: To what extent do demographic and professional factors (such as gender, age, staff position, and education) predict posttest perceived handover competency (PC)?
 RQ3: To what extent do internal psychological factors (IIH, ICH, RH, and PP) predict posttest perceived handover competency (PC)?

Based on the theoretical framework and the objectives of this study, we proposed the following hypotheses:

 H1: The CARE protocol‐related simulation‐based training will lead to a statistically significant increase in nurses’ perceived handover competency (PC) from pretest to posttest.
 H2: Demographic and professional factors (gender, age, staff position, and education) will significantly predict post‐test perceived handover competency (PC).
 H3: Internal psychological factors (IIH, ICH, RH, and PP) will significantly predict posttest perceived handover competency (PC), independently contributing to the variance explained beyond demographic and professional variables.

By identifying patterns in competency development, this study provides empirical evidence to inform targeted educational designs and policy initiatives. Ultimately, this study aims to improve clinical communication and patient safety in Hong Kong and similar global contexts.

## 2. Methods

### 2.1. Aim

This study aimed primarily to evaluate the effect of simulation‐based handover training on nurses’ perceived handover competency. A secondary aim was to identify demographic and psychological factors that predict nurses’ perceived handover competency.

### 2.2. Design

A single‐group, quasi‐experimental, pretest–posttest intervention design was employed. Reporting follows the Transparent Reporting of Evaluations with Nonrandomized Designs (TREND) statement to ensure methodological transparency. The TREND statement checklist is available in Supporting File S1.

### 2.3. Setting and Participants

Participants were recruited between January 2023 and March 2023 using a two‐stage sampling strategy. In the first stage, cluster sampling was employed by identifying and inviting 29 public hospitals (managed by the Hospital Authority) in Hong Kong to participate. In the second stage, purposive sampling was used to recruit individual nurses from these hospitals who met the following inclusion criteria: (1) held a valid nursing qualification; (2) were proficient in English; and (3) provided written consent. According to established criteria for correlation, at least 111 participants are required to detect a strong correlation (*r*_*s*_ ≥ 0.50) with a 95% confidence interval [22]. For the subsequent OLS regression analysis, standard methodological formulas require a sample size of at least 195 cases [23]. Our final sample of 501 nurses exceeded all thresholds, yielding a robust subject‐to‐predictor ratio and ensuring strong statistical power. These participants were drawn from a range of clinical units, including acute care, surgical wards, and intensive care units.

### 2.4. Instruments

The training materials included guidelines for implementing the CARE protocol during handovers, guidelines for simulation sessions, and notes for peer feedback. Data collection was facilitated through a separate, multidimensional questionnaire, the details of which are provided in Supporting File S2. This questionnaire was divided into three distinct sections to collect (1) demographic and professional data, (2) internal psychological data and evaluative outcomes, and (3) feedback for the training.

### 2.5. Section A: Demographic and Professional Characteristics

This section collected demographic and professional data from the participating nurses. The variables included age, gender, education level (e.g., biploma, bachelor’s, and master’s), current staff position (e.g., enrolled, nurse, registered nurse [RN], and advanced practice nurse [APN]), and prior exposure to formal handover training.

### 2.6. Section B: Internal Psychological Factors and Perceived Competency

This section evaluated the psychological (IIH, ICH, RH, and PP) and behavioral outcomes (PC) of the simulation‐based training using 5‐point Likert scales, ranging from 1 *(strongly disagree)* to 5 *(strongly agree)*.

#### 2.6.1. Internal Psychological Factors

Internal psychological factors were evaluated using a 17‐item instrument theoretically grounded in Kuhl’s action control theory. In constructing this tool, we referred to the psychometric scales reported by Lee and Lim [15] (Cronbach’s *α* = 0.958). However, the items were recontextualized and modified by the research team to ensure strict alignment with the conceptual domains of Kuhl’s action control theory. All 17 items were rated on a five‐point Likert scale from 1 *(strongly disagree)* to 5 *(strongly agree)*. The instrument comprises four distinct subscales to measure psychological factors:

1. Perceptions of protocols (PP) measure the perceived utility outcome using two items adapted from a validated questionnaire assessing the clinical value of standardized handover tools.
2. IIH, 4 items, and ICH, 3 items, measure selective motivation outcomes following the training.
3. RH, 8 items, measures the volitional control outcome of the nursing staff under simulated pressure.

#### 2.6.2. Perceived Handover Competency

The primary outcome of the intervention, perceived handover competency, was measured using an 11‐item instrument adapted from the CARE protocol‐related questionnaire, which was originally used in a previous study in Hong Kong hospitals [24] and demonstrated a Cronbach’s *α* of 0.99. Since this questionnaire was previously developed by our research team, formal permission for its reuse in this study was granted and retained by the authors. Each of the 11 items was rated on a 5‐point Likert scale, ranging from 1 *(strongly disagree)* to 5 *(strongly agree)*.

#### 2.6.3. Instrument Validation

To ensure clinical relevance and content validity across both instruments, an expert panel comprising two nursing professors and three advanced practice nurses, each with over 6 years of experience, was convened. The panel reviewed all 28 items to ensure they accurately captured the communicative nuances of the CARE protocol and the psychological constructs of action control theory within the specific context of the handover environment. Following this review, items were refined for linguistic clarity and clinical applicability to the simulation‐based training intervention.

The internal consistency (reliability) for each subscale in the current sample was high, exceeding the recommended threshold of 0.7 for nursing research. Details are presented in Table 1.

**TABLE 1:** Internal consistency and item–total correlation of the five‐factor handover communication scale (*n* = 501).

Factor	Number of items	CITC range	Cronbach’s *α*
Intention to initiate handover (IIH)	4	0.584–0.712	0.845
Intention to complete handover (ICH)	3	0.621–0.745	0.812
Resilience in handover (RH)	8	0.556–0.789	0.910
Perceptions of protocol (PP)	2	0.612–0.612	0.768
Perceived handover competency (PC)	11	0.590–0.811	0.932
Total scale	28	0.556–0.811	0.954

*Note:* Cronbach’s *α* > 0.7 indicates good internal consistency. CITC values > 0.5 indicate that the item is highly consistent with other items within its corresponding subscale.

Abbreviation: CITC: corrected item–total correlation.

The total scale demonstrated excellent internal consistency with an overall Cronbach’s *α* of 0.954. By analyzing these as separate constructs, the study was able to determine the unique variance each domain contributed to the overall improvement in handover performance.

### 2.7. Section C: Training Feedback

The third section collected qualitative and quantitative feedback regarding the simulation‐based workshop, including the perceived relevance of the materials and the quality of the facilitators.

### 2.8. Intervention

**FIGURE 1:**
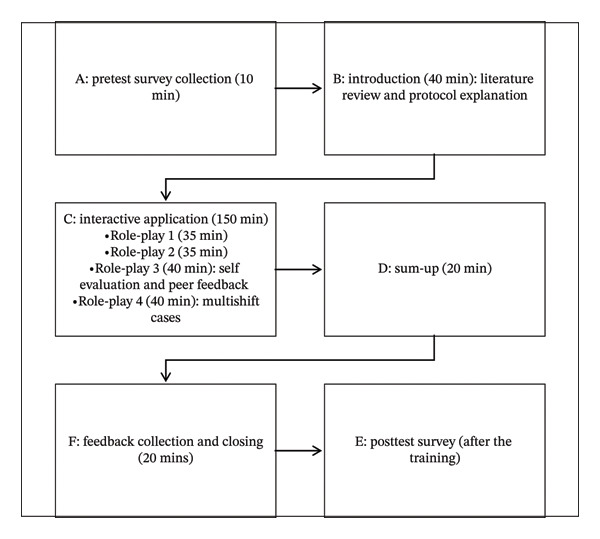
Flowchart of workshop.

The intervention was a 4‐h offline communication training delivered in local hospital seminar rooms across 15 sessions between March 2023 and May 2023 (see Figure 1). A pilot test involving 14 participants was conducted in August 2022 to refine the curriculum and procedures [24]. Each session was capped at 40 to 42 nurses, which maintained a low facilitator‐to‐participant ratio of 1:7 to ensure practical small‐group discussion, personalized feedback, and interactive learning. The study lead, who has expertise in nursing handover training, delivered the training with the help of facilitators. The allocation to each group was determined based on a first‐come, first‐served basis. Guided by Madeline Hunter’s direct instruction model, the intervention used a structured pedagogical approach to facilitate skill acquisition [25]. A comprehensive breakdown of the instructional stages is provided in Supporting File S3.

### 2.9. Facilitator Training and Fidelity Monitoring

To ensure consistent content delivery across the 15 sessions, all workshops were conducted by the study lead and trained facilitators. Before the official launching of the workshops, six facilitators completed a one‐hour standardized training program conducted by the study lead that covered the workshop learning objectives, CARE materials, role‐play scripts, debriefing procedures, and the use of the facilitator guide. To monitor fidelity, we used a two‐step approach. First, after each workshop, the facilitator completed a standardized facilitator guide to confirm coverage of the required components (learning objectives, video demonstration, role plays, guided reflection, and distribution of learning materials). Second, an independent fidelity assessor attended four randomly selected sessions and completed the same checklist. The assessor’s ratings were then compared with the facilitators’ self‐reports. Across the assessed sessions, fidelity to core elements averaged 89%. Deviations, most commonly in time allocation between role‐play and discussion, were used to adjust subsequent facilitator briefing notes to improve the standardization of the workshops.

The workshop was structured around the CARE protocol, which is a widely recognized tool for enhancing the quality and teamwork of clinical handovers [6, 7]. The training included interactive components such as video demonstrations (developed by the research team in collaboration with the facilitators), structured role‐plays, case‐based discussions, and guided reflection [19].

### 2.10. Data Collection

Participants completed the pretraining assessment (T0) immediately before the 4‐h training session. The posttraining evaluation (T1) was administered immediately after the workshop concluded. Data were collected in hospital seminar rooms before and after training. The same instrument was used at both times to assess change across the five constructs. 501 participants (89.8%) completed both the pre‐ and posttest questionnaires, providing valid data for analysis. All participants provided written informed consent before the intervention.

### 2.11. Statistical Analysis

Descriptive statistics, including frequencies, percentages, means, and standard deviations, were used to summarize the demographic characteristics and scores for each factor (IIH, ICH, RH, PP, and PC). While individual Likert items are ordinal, they were treated as continuous interval variables when aggregated into composite scores (IIH, ICH, RH, PP, and PC). This approach aligns with established practices in medical education research, which recognizes that the summation of Likert‐type responses provides a continuous‐like distribution suitable for parametric analysis [26, 27]. The normality of these aggregate distributions was assessed using the Kolmogorov–Smirnov test, skewness, and kurtosis coefficients. Although formal normality tests were significant given the large sample size (*n* = 501), descriptive statistics of skewness (± 0.43) and kurtosis (< 1.5) confirmed that aggregate scores were approximately normally distributed. Consequently, parametric tests were employed as the statistical methods.

The effect of the intervention on PC was evaluated using paired‐samples *t*‐tests to compare pretest and posttest scores. This was complemented by Cohen’s *d* to quantify and interpret the effect size. Differences in aggregate scores across demographic categories were evaluated using paired‐samples *t*‐tests and one‐way ANOVA.

Correlations between internal psychological factors and PC were evaluated using Pearson’s correlation (*r*). We verified that the data met the requirements for continuous measurement (aggregated scores) and linear association before this analysis. To identify independent predictors of the effect of training, a two‐stage ordinary least squares (OLS) regression was conducted using posttest aggregate scores. Categorical variables, such as staff position, were dummy‐coded before data entry. Model 1 included demographic and professional baseline variables, while Model 2 incorporated internal psychological domains (ICH, RH, IIH, and PP). The incremental variance explained by the psychological factors (Δ*R* ^2^) was evaluated to assess the robustness of these predictors beyond baseline characteristics. Multicollinearity was assessed using variance inflation factors (VIFs), with values smaller than 10 considered acceptable. All analyses were two‐tailed, with statistical significance set at *p* < 0.05.

### 2.12. Ethical Considerations

The study protocol was approved by the Ethics Committee of the City University of Hong Kong (Ref: HU‐STA‐00000532). All participants provided written informed consent before data collection.

## 3. Results

### 3.1. General Findings

**FIGURE 2:**
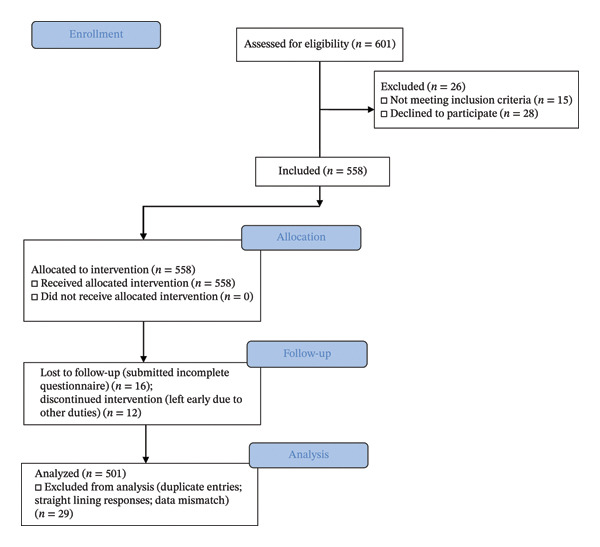
Flowchart of participants.

A total of 501 nurses completed the study, of whom 76.4% (*n* = 383) were female, and the majority (82.8%) were aged between 20 and 29 years. Most participants were enrolled nurses (85.2%) and held a bachelor’s degree (70.3%). Only 13% of the sample had received prior training related to clinical handovers. Figure 2 shows the participant flow of the study.

Single‐factor analysis is shown in Table 2. Paired‐samples *t*‐tests were used for dichotomous variables (gender and prior training), while one‐way ANOVA was used for variables with more than two groups (age group, staff position, and education level). The analysis revealed statistically significant differences in the standardized growth of perceived competency scores based on age group (*F* = 3.204, *p* = 0.023) and staff position (*F* = 3.813, *p* = 0.023). No significant differences in score growth were observed for gender (*p* = 0.599), prior training (*p* = 0.168), or education level (*p* = 0.416). Across all demographic categories, mean scores improved from pretest to posttest, with the highest posttest scores observed in the ≥ 35 age group (*M* = 3.709, SD = 0.542) and among advanced practice nurses (*M* = 3.724, SD = 0.540).

**TABLE 2:** Single factor analysis (*n* = 501).

Factor	Category	*n* (%)	Pretest score (*M* ± *SD*)	Posttest score (*M* ± *SD*)	*t*/*F* value	*p* value
Gender	Male	118 (23.6%)	2.848 ± 0.451	3.409 ± 0.456	*t* = 0.525	0.599
	Female	383 (76.4%)	2.819 ± 0.423	3.352 ± 0.510		

Age group	20–24	200 (39.9%)	2.735 ± 0.337	3.253 ± 0.398	*F* = 3.204	0.023
	25–29	215 (42.9%)	2.813 ± 0.415	3.373 ± 0.516		
	30–34	31 (6.2%)	2.689 ± 0.398	3.437 ± 0.587		
	≥ 35	55 (11.0%)	3.284 ± 0.514	3.709 ± 0.542		

Staff position	Registered nurse	24 (4.8%)	2.689 ± 0.383	3.345 ± 0.434	*F* = 3.813	0.023
	Enrolled nurse	427 (85.2%)	2.772 ± 0.383	3.325 ± 0.480		
	Advanced practice nurse	50 (10.0%)	3.353 ± 0.472	3.724 ± 0.540		

Prior training	No	436 (87.0%)	2.780 ± 0.405	3.331 ± 0.493	*t* = 1.38	0.168
	Yes	65 (13.0%)	3.136 ± 0.462	3.597 ± 0.473		

Education level	Diploma	76 (15.2%)	2.696 ± 0.396	3.292 ± 0.405	*F* = 0.879	0.416
	Bachelor	352 (70.3%)	2.799 ± 0.395	3.338 ± 0.487		
	Master’s or above	73 (14.6%)	3.090 ± 0.515	3.579 ± 0.583		

### 3.2. Effect of Simulation‐Based Training on Perceived Handover Competency

The overall efficacy of the intervention was evaluated by comparing pretest and posttest aggregated PC scores using a paired samples *t*‐test. Results revealed a statistically significant improvement (*t* = 26.813, *df* = 500, *p* < 0.001), with the aggregated PC score increasing from pretest (*M* = 2.776, *SD* = 0.406) to posttest (*M* = 3.409, *SD* = 0.494). Effect sizes were interpreted according to Cohen’s guidelines, where *d* = 0.2 indicates a small effect, 0.5 a medium effect, and 0.8 a large effect [28]. The mean difference was 0.633 (95% CI [0.587, 0.679]) with a large effect size (Cohen’s *d* = 1.198). PC had an overall growth rate of 22.84%, indicating that simulation‐based training effectively enhanced nurses’ perceived capabilities in performing handovers.

The ranking of PC scores by growth rate is shown in Table 3. In terms of individual item performance, the posttest perceived competency scores revealed a clear hierarchy between technical execution and internal confidence. The highest‐ranked competencies were predominantly task‐oriented, led by Item 18 (“I am confident in my ability to perform a systematic handover of the patient’s identity, room number, and diagnosis”), followed closely by Item 23 on the reason for hospitalization and Item 20 on handovers following abnormal vital‐sign changes. Conversely, the lowest scores were for the psychological aspects of the task, specifically Items 15 (“I trust myself to implement protocols [like CARE] in handovers”) and 14 (“I am confident in my ability to perform a handover”). This pattern suggests that while the intervention effectively strengthened the nurses’ perceived ability to communicate clinical data, it had a comparatively smaller impact on their overall trust in their handover performance.

**TABLE 3:** Ranking of perceived competency scores of nurses (*n* = 501).

Rank	Item	Description	Pretest (*M* ± *SD*)	Posttest (*M* ± *SD*)	Growth rate (%)
1	Q18	I am confident in my ability to perform a systematic handover of the patient’s identity, room number, and diagnosis.	2.715 ± 0.751	3.643 ± 0.760	34.19%
2	Q23	I can clearly explain the clinical reason for hospitalization.	2.739 ± 0.664	3.585 ± 0.715	30.90%
3	Q20	I am confident in recognizing and reporting abnormal changes in vital signs or omitting non‐essential data during transitions.	2.784 ± 0.682	3.487 ± 0.659	25.23%
4	Q19	I can accurately communicate the patient’s primary complaint and objective clinical outcomes.	2.733 ± 0.684	3.421 ± 0.552	25.20%
5	Q25	I am confident in my ability to perform a handover of specific actions taken and future nursing care plans.	2.681 ± 0.628	3.327 ± 0.534	24.13%
6	Q24	I can perform a handover of medications taken, allergies, and other medication‐specific conditions.	2.876 ± 0.601	3.429 ± 0.634	19.22%
7	Q21	I am proficient in delivering detailed explanations of treatments, test results, and relevant patient charts.	2.814 ± 0.576	3.355 ± 0.574	19.22%
8	Q26	I can perform a handover while addressing the patient’s and caregiver’s emotional needs.	2.896 ± 0.581	3.445 ± 0.663	18.95%
9	Q22	I can perform a handover with detailed explanations of the patient’s risks and falls.	2.856 ± 0.543	3.395 ± 0.606	18.87%
10	Q14	I am confident in my ability to perform a handover.	2.669 ± 0.581	3.168 ± 0.566	18.70%
11	Q15	I trust myself to implement protocols (like CARE) in handovers.	2.768 ± 0.568	3.248 ± 0.501	17.30%

### 3.3. Correlation Between Perceived Competency and Psychological Factors

The relationship between PC and psychological factors was evaluated using Pearson’s correlation analysis. As shown in Table 4, the results demonstrated that PC was significantly and positively correlated with all measured domains (*p* < 0.001). The strongest correlations were observed between PC and RH (*r* = 0.920, *p* < 0.001) and between PC and IIH (*r* = 0.895, *p* < 0.001). PC also demonstrated strong correlations with ICH (*r* = 0.876, *p* < 0.001) and PP (*r* = 0.808, *p* < 0.001). These findings suggest that nurses who showed higher levels of resilience and greater willingness to initiate handovers also reported higher perceived competence in their practice.

**TABLE 4:** Pearson’s correlation analysis (*n* = 501).

Factor	IIH	ICH	RH	PP	PC
Intention to initiate handover (IIH)	1	0.892^∗∗∗^	0.912^∗∗∗^	0.738^∗∗∗^	0.895^∗∗∗^
Intention to complete handover (ICH)	0.892^∗∗∗^	1	0.892^∗∗∗^	0.732^∗∗∗^	0.876^∗∗∗^
Resilience in handover (RH)	0.912^∗∗∗^	0.892^∗∗∗^	1	0.768^∗∗∗^	0.920^∗∗∗^
Perception of protocol (PP)	0.738^∗∗∗^	0.732^∗∗∗^	0.768^∗∗∗^	1	0.808^∗∗∗^
Perceived competency (PC)	0.895^∗∗∗^	0.876^∗∗∗^	0.920^∗∗∗^	0.808^∗∗∗^	1

^∗∗∗^ *p* < 0.001.

### 3.4. Predictors of Improvement in Perceived Competency

To identify the independent predictors of perceived improvement in competency, an OLS regression analysis was conducted using two models. As shown in Table 5, Model 1, which included only demographic variables (gender, age, position, training, and education), explained a small portion of the variance (*R* ^2^ = 0.018, *p* > 0.05), falling well below Cohen’s (1988) threshold for a small effect (*R* ^2^ = 0.02) [28]. Including the internal psychological factors (Model 2) significantly enhanced the model’s explanatory power, accounting for 76.5% of the total variance (*R* ^2^ = 0.765, *F* = 177.57, *p* < 0.001). Within this full model in Table 6, staff position remained a significant demographic predictor (*β* = −0.063, *p* = 0.044), while all four internal domains were found to be highly significant: IIH (*β* = 0.110, *p* = 0.002), ICH (*β* = 0.320, *p* < 0.001), RH (*β* = 0.376, *p* < 0.001), and PP (*β* = 0.202, *p* < 0.001). Notably, resilience in handover (RH) was the most influential factor (*β* = 0.376, *p* < 0.001), suggesting that resilience and persistence are the most important determinants of a nurse’s perceived growth in competency after training.

**TABLE 5:** Explained variance in perceived handover competency.

Variable	Model 1 (demographics)	Model 2 (full model)
*R* ^2^ (explained variance)	0.018	0.765^∗∗∗^

*Note:* Model 1 includes only demographic and professional variables. Model 2 adds internal psychological factors (IIH, ICH, RH, and PP). *R* ^2^ values of 0.02, 0.13, and 0.26 represent small, medium, and large effects, respectively [28].

^∗∗∗^ *p* < 0.001.

**TABLE 6:** OLS regression analysis of factors influencing perceived competency.

Variable	*B*	*SE*	*β*	*t*	*p* values	VIF
(Constant)	0.167	0.106	—	1.575	0.116	—
Gender	0.031	0.040	0.017	0.778	0.437	1.019
Age group	0.041	0.023	0.050	1.805	0.072	1.573
Staff position	−0.127	0.063	−0.063	−2.021	0.044^∗^	2.007
Prior training	−0.045	0.055	−0.019	−0.806	0.421	1.200
Education	0.000	0.037	0.000	−0.011	0.991	1.436
IIH score	0.112	0.036	0.110	3.139	0.002	2.571
ICH score	0.303	0.035	0.320	8.631	^∗∗∗^	2.863
RH score	0.412	0.045	0.376	9.143	^∗∗∗^	3.540
PP score	0.168	0.022	0.202	7.796	^∗∗∗^	1.397

*Note:* VIF values were all below 5, indicating no significant multicollinearity among the independent variables.

^∗^ *p* < 0.05.

^∗∗^ *p* < 0.01.

^∗∗∗^ *p* < 0.001.

## 4. Discussion

This study evaluated the efficacy of a simulation‐based handover training program within the Hong Kong clinical landscape. Our findings demonstrate that while the intervention effectively enhanced technical proficiency, there remains significant room for fostering psychological readiness. Furthermore, we identified that while professional experience provides a higher baseline of competence, resilience acts as the primary driver of training receptivity and clinical application.

### 4.1. RQ1: Effect of Simulation‐Based Training on Perceived Handover Competency

This study indicates that the intervention improved overall perceived competency, underscoring the efficacy of simulation‐based training in bridging the gap between theoretical knowledge and clinical practice. This finding is consistent with nursing literature from Western settings, such as Australia and the United Kingdom, which confirms that simulation‐based education enhances technical proficiency and confidence during handovers [5, 9]. While most existing research focuses on the ISBAR framework in Western health systems, this study validates the efficacy of the CARE protocol in Hong Kong’s clinical landscape. Our findings add to the existing literature showing that the fundamental principles of standardized handover communication are applicable, regardless of the specific protocol or cultural context [2, 8, 24].

A granular analysis of item rankings reveals a distinct divergence between technical proficiency and psychological self‐assurance. Participants demonstrated high levels of confidence in task‐oriented, objective data transfer, especially for patient identifiers (Item 18), reasons for admission (Item 23), and reporting abnormal vital signs (Item 20). These elements align with the “clinical status and condition” and “actions and assessment” components of the CARE protocol, which are concrete, prescriptive, and less subject to interpersonal variability.

In contrast, the lowest scores were consistently observed in items related to internal self‐trust and professional readiness (Items 14 and 15). This suggests that while a 4‐h training workshop may be sufficient to teach the mechanics of a protocol, fostering confidence in one’s ability to apply handovers remains a challenge. While the intervention improved baseline scores, the findings indicate that integrating standardized protocols into autonomous clinical practice entails more than rote memorization [29]. For nurses to have stronger self‐trust and professional readiness to execute protocols in high‐stakes environments, they may require longitudinal mentorship and consistent feedback loops that enhance nurses’ communicative self‐efficacy [30].

### 4.2. RQ2: Role of Demographic and Professional Factors in Predicting Perceived Competency

To identify the influence of individual background on these outcomes, we utilized single‐factor analysis and OLS regression. Demographic and professional factors exert only a marginal influence on the overall growth of handover competency following simulation‐based training. The single‐factor analysis revealed that growth in perceived competency differed significantly based on age group and staff position.

In alignment with existing competency development literature [11], staff position was a significant predictor of PC (*p* = 0.044). APNs and nurses aged 35 years or older consistently achieved the highest absolute scores, likely benefiting from the crystallized intelligence and situational awareness cultivated through long‐term clinical experience [31]. However, a notable finding was the optimal receptivity observed among RNs and those aged 30 to 34 years. While senior nurses maintained higher absolute proficiency, younger RNs showed a more pronounced growth rate following the simulation‐based training. Within Kuhl’s framework, this pattern may reflect a difference in the balance between selective motivation and volitional control. Younger RNs may have greater motivational readiness to adopt new protocols (higher IIH and ICH), whereas senior staff draw on well‐established volitional routines developed through clinical experience. This aligns with previous findings that early‐career nurses possess sufficient foundational knowledge to grasp the clinical stakes of handover while maintaining the cognitive plasticity required to integrate new protocols [8].

### 4.3. RQ3: Relationship Between Perceived Competency and Psychological Factors

To explore the extent to which internal psychological factors (ICH, IIH, RH, and PP) predict post‐test PC, we synthesized findings from both our Pearson correlation analysis and the OLS regression. The OLS regression analysis (Model 2) shows that these psychological domains collectively account for 76.5% of the variance in competency growth. The strong predictive power of psychological factors is consistent with Kuhl’s Action Control Theory, which holds that behavioral outcomes are determined not by experience alone but by the interplay between selective motivation (operationalized here as IIH and ICH) and volitional control (operationalized as RH). A nurse’s perceived handover competency, in this view, reflects the degree to which both stages of the action control cycle—selective motivation and volitional control—are successfully engaged.

The correlation analysis revealed that RH had the strongest positive relationship with perceived handover competency (PC) among all internal psychological factors examined (*r* = 0.920, *p* < 0.001). This finding suggests that perceived handover competency is not simply an outcome of accumulated clinical experience but is influenced by a nurse’s capacity to protect their communicative intentions from real‐world interference. This is broadly consistent with a growing body of literature identifying resilience as a factor in positive behavioral change in clinical settings [32–35]. However, despite its essential role in general nursing practice, resilience has received limited attention as a discrete construct in nursing handover research, where self‐efficacy and protocol adherence have typically been the factors measured [10, 16]. Consequently, this finding about resilience points to a clear direction for nursing workforce development. Hospital administrators should implement targeted interventions that nurture resilience in handover training. Strengthening these internal volitional drivers may be the key to ensuring that standardized protocols move from mere checklists to internalized professional standards.

### 4.4. Limitations and Future Research

This study has several limitations that should be considered when interpreting the results. First, the reliance on self‐reported questionnaires may not fully capture actual handover behaviors in clinical practice and is susceptible to social desirability bias. Second, the quasiexperimental pretest–posttest design lacked a control group, making it difficult to attribute all observed changes solely to the intervention. Third, as is common in educational research, blinding of participants and facilitators was not feasible, which may have influenced self‐assessment responses. These limitations directly inform valuable avenues for future research. Longitudinal studies with extended follow‐up periods are needed to assess the sustainability of training effects and their translation into routine practice. Furthermore, mixed‐method approaches that supplement self‐report with direct observations, audio recordings of handovers, and feedback from receiving nurses could provide richer, more objective insights into communication behaviors and the true impact of training on clinical outcomes. Future studies employing RCT designs would also help strengthen causal inferences regarding the efficacy of tailored training modules.

## 5. Conclusion

This study demonstrates that simulation‐based training using the CARE protocol effectively enhances nursing handover competency. Our findings show that perceived competency is mainly influenced by internal psychological factors rather than demographic factors. Psychological factors accounted for 76.5% of the variance in outcomes, whereas professional factors explained only 1.8%. Resilience was particularly important, serving as the volitional control for effective communication under high‐stakes conditions and acting as the strongest predictor of perceived competency. We recommend that healthcare organizations shift their focus from implementing rote checklists to longitudinal, simulation‐based training that integrates resilience‐building strategies. This approach can foster a safety culture and ensure that standardized communication protocols are internalized as essential clinical standards.

**Nomenclature**
APN: Advanced practice nurse
CARE: Connect, Ask, Respond, and Empathize
EN: Enrolled nurse
ICH: Intention to complete handover
IIH: Intention to initiate handover
PC: Perceived handover competency
PP: Perceptions of protocol
RH: Resilience in handover
RN: Registered nurse

## Author Contributions

Jack Pun designed the study, wrote the research protocol, and revised the manuscript; Kexin Tan conducted the literature review and quality control; collected, checked, and analyzed the data; and prepared the manuscript.

## Funding

No funding was received for this research.

## Disclosure

All authors read and approved the final manuscript.

## Ethics Statement

This study was approved by the ethics committee of the City University of Hong Kong (Ref: HU‐STA‐00000532).

## Consent

All participants provided informed consent before participating in the study.

## Conflicts of Interest

The authors declare no conflicts of interest.

## Supporting Information

Additional supporting information can be found online in the Supporting Information section.

## Supporting information


**Supporting Information 1** Supporting File S1: TREND checklist of the study.

**Supporting Information 2** Supporting File S2: Details about the questionnaire used in this study.

**Supporting Information 3** Supporting File S3: The instructional stages of the intervention guided by Madeline Hunter’s model.

## Data Availability

The datasets used and/or analyzed during the current study are available from the corresponding author on reasonable request.
