# Supplementary material for: Evaluating the Effect of Simulation‐Based Handover Training and Its Predictors: A Quasiexperimental Study
Source: J Nurs Manag. 2026 Jul 29;2026:6626003. doi: 10.1155/jonm/6626003 (PMC13420279; doi:10.1155/jonm/6626003)
Supplement: Supplementary file 2 — Supporting Information 2 Supporting File S2: Details about the questionnaire used in this study. [file JONM-2026-6626003-s003.docx]

**Supplementary File S2**

| Construct | Definition | Item Numbers |
| --- | --- | --- |
| Intention to Initiate Handover (IIH) | Measures readiness and self-belief in starting handovers under varied clinical conditions. | 1, 2, 8, 9 |
| Intention to Complete Handover (ICH) | Captures persistence and commitment to follow-through until tasks are fully accomplished. | 4, 5, 16 |
| Resilience in Handover (RH) | Assesses hardiness (Commitment, Control, Challenge) and determination when facing adversities. | 3, 6, 7, 10, 11, 12, 13, 17 |
| Perceptions of Protocols (PP) | Evaluates attitudes toward structured tools (e.g., CARE/ISBAR), including safety benefits and barriers. | 27, 28, 29, 30 |
| Perceived Competency (PC) | Reflects self-efficacy in applying protocols to perform accurate and meaningful clinical handovers. | 14, 15, 18, 19, 20, 21, 22, 23, 24, 25, 26 |

**Construct 1: Intention to Initiate Handover (IIH)**

Item 1. I believe I can perform a handover whenever I plan to.

Item 2. I can perform a handover if required.

Item 8. I can perform an unpleasant handover if I am required to.

Item 9. I can perform a sudden handover if a patient has to be taken to the ward.

**Construct 2: Intention to Complete Handover (ICH)**

Item 4. I can accomplish the goal of a handover.

Item 5. I do not give up on any task and ensure I complete it (handover).

Item 16. I do not give up while performing a handover.

**Construct 3: Resilience in Handover (RH)**

Item 3. I can work hard even when it is difficult to start a handover.

Item 6. I can attempt a difficult handover.

Item 7. I can attempt a complicated handover.

Item 12. I try to learn even if the handover seems difficult.

Item 10. Even if I fail at first, I do not give up when learning something new.

Item 13. I am not frustrated when I fail to perform a handover and try to work even harder.

Item 11. I can handle unexpected events during a handover.

Item 17. I can handle any problems that may arise during a handover.

**Construct 4: Perceptions of Protocols (PP)**

Item 27. I believe using CARE will help me improve my communication skills with my co-workers.

Item 28. I believe using CARE will improve patient quality and safety.

**Construct 5: Perceived Competency (PC)**

Item 14. I am confident in my ability to perform a handover.

Item 15. I trust myself to implement protocols (like CARE) in handovers.

Item 18. I am confident in my ability to perform a systematic handover of the patient’s identity, room number, and diagnosis.

Item 19. I can accurately communicate the patient’s primary complaint and objective clinical outcomes.

Item 20. I am confident in recognizing and reporting abnormal changes in vital signs or omitting non-essential data during transitions.

Item 21. I am proficient in delivering detailed explanations of treatments, test results, and relevant patient charts.

Item 22. I can perform a handover with detailed explanations of the patient’s risks and falls.

Item 23. I can clearly explain the clinical reason for hospitalization.

Item 24. I can perform a handover of medications taken, allergies, and other medication-specific conditions.

Item 25. I am confident in my ability to perform a handover of specific actions taken and future nursing care plans.

Item 26. I can perform a handover while addressing the patient’s and caregiver’s emotional needs.
