# Supplementary material for: Evaluating the Effect of Simulation‐Based Handover Training and Its Predictors: A Quasiexperimental Study
Source: J Nurs Manag. 2026 Jul 29;2026:6626003. doi: 10.1155/jonm/6626003 (PMC13420279; doi:10.1155/jonm/6626003)
Supplement: Supplementary file 3 — Supporting Information 3 Supporting File S3: The instructional stages of the intervention guided by Madeline Hunter’s model. [file JONM-2026-6626003-s001.docx]

**Supplementary File S3**

The intervention was based on Madeline Hunter’s direct instruction model

| Step | Application | Component |
| --- | --- | --- |
| 1.      Anticipatory Set: Provide a hook for students to see the importance and relevance of learning | Online Module: A video of a failed handover resulting in a patient near-miss. | *Engagement* |
| 2.      Objective: Identify what students will be able to do, understand, and/or care about as a result of the lesson | Online: The utilization of the CARE protocol in clinical settings. | *Informational* |
| 3.      Teaching & Modeling: Provide a model or example of what the expected outcome of the learning is | Online: Demonstration videos showing a Good vs. Bad handover. | *Informational* |
| 4.      Checking for Understanding: Evaluate students’ practice sets | Online: Knowledge quizzes on the acronyms. | *Interactional* |
| 5.      Guided Practice: Students work on activities relevant to the subject matter | Simulation: A scenario role-play in which facilitators provide live feedback. | *Interactional* |
| 6.      Independent Practice: Have students repeat the practice to reinforce the learning | Simulation: Handover with different scenarios (e.g., houseman or MO). | *Interactional* |
| 7.      Closure: Actions or words by the teacher that provide cues to students that they have learned the subject matter | Debriefing: Reflective learning session to summarize areas for improvement. | *Synthesis* |
